# A Novel Model of Ultrasonic Fatigue Test in Pure Bending

**DOI:** 10.3390/ma15144864

**Published:** 2022-07-13

**Authors:** Dongtong Yang, Sen Tang, Yongtao Hu, Alexander Nikitin, Qingyuan Wang, Yongjie Liu, Lang Li, Chao He, Yan Li, Bo Xu, Chong Wang

**Affiliations:** 1 MOE Key Laboratory of Deep Earth Science and Engineering, College of Architecture and Environment, Sichuan University, Chengdu 610065, China; ydt19961221@foxmail.com (D.Y.); yongthu@163.com (Y.H.); wangqy@scu.edu.cn (Q.W.); liuyongjie@scu.edu.cn (Y.L.); lilang@scu.edu.cn (L.L.); hechao@scu.edu.cn (C.H.); lileah2016@scu.edu.cn (Y.L.); 2 School of Architecture and Civil Engineering, Chengdu University, Chengdu 610106, China; tangsen@stu.cdu.edu.cn; 3 Institute for Computer Aided Design, Russian Academy of Science, 123056 Moscow, Russia; nikitin_alex@bk.ru; 4 Failure Mechanics and Engineering Disaster Prevention and Mitigation Key Laboratory of Sichuan Province, Sichuan University, Chengdu 610207, China

**Keywords:** very high cycle fatigue, experimental method, flexural vibration, thin plate material

## Abstract

The very high cycle fatigue (VHCF) failure of in-service components is mainly caused by the vibration of thin-wall elements at a high frequency. In this work, a novel model of ultrasonic fatigue test was developed to test thin-wall material in bending up to VHCF with an accelerated frequency. The theoretical principle and finite element analysis were introduced for designing a sample that resonated at the frequency of 20 kHz in flexural vibration. In the advantage of the second-order flexural vibration, the gauge section of the sample was in the pure bending condition which prevented the intricate stress condition for thin-wall material as in the root of cantilever or the contact point of three points bending. Moreover, combining the constraint and the loading contact in one small section significantly reduced heating that originated from the friction at an ultrasonic frequency. Both strain gauge and deflection angle methods were applied to verify the controlling of stress amplitude. The fractography observation on Ti6Al4V samples indicated that the characterized fracture obtained from the novel model was the same as that from the conventional bending test.

## 1. Introduction

Piles of mechanical structures are subject to high-frequency with small-amplitude variable loadings in aviation, aerospace, and other industrial fields. However, with the increased reliability requirements of centrifugal compressors, aero-engines, and other pieces of equipment, the low cycle fatigue (LCF)/high cycle fatigue (HCF) theory and analysis methods are dubious when directly applied to predicting the fatigue life and strength of mechanical components in a very high cycle fatigue (VHCF) regime [1]. The study on the VHCF plays an important role in the last decade because of the development of the ultrasonic fatigue test instrument that allows the specimens to reach a very high cycle in a reasonable time owing to its high frequency. Considering time consumption alone, it is almost impossible to utilize traditional experimental methods for this research field [2]. Hence, in 1911, Hopkinson [3], Jenkin and Lemann [4] first studied the problem of high-frequency fatigue. In 1950, Mason [5] used a piezoelectric crystal to convert the electromagnetic wave signal of 20 kHz into mechanical vibration, developing the first set of test equipment that could vibrate and deform the sample at a frequency of 20 kHz. Since the 1970s, this high-frequency vibration, ultrasonic fatigue test technique (f=15–22 kHz), based on the principle of piezo-electromagnetic strain, has been applied to the study of fatigue fracture of materials [6]. Afterwards, several devices using the ultrasonic testing technique have been developed all over the world, especially since the end of the last century [7].

During the complex service condition, the thin sheet components, such as the blades in an engine, are likely subjected to an unexpected vibration, bringing the greatest harmfulness in service. It was found that [7,8] the blade vibration frequency could be increased by the centrifugal force field, and the blade resonance frequency could be increased more significantly by prominent dynamic stiffness affection. Thus, improving the evaluation of fatigue life in the vibration mode at the resonance frequency could significantly benefit the durability design of structural integrity.

Although the fatigue data from either axial loading or vibration deformation indicate the material property, the difference caused by test methods is often reported for precise analysis of fatigue behavior. In bending fatigue, maximum stresses are generated on the surface of the specimen, where a fatigue crack is expected to be initiated. In contrast, axial fatigue tests generate uniform stresses on the cross-section of fatigue specimens and, consequently, subsurface defects can also initiate a fatigue crack. A comparison of the test results for axial loading and rotary bending shows a higher scatter of results for the latter. A higher scatter of results under rotary bending can be explained by the effect of material structure non-uniformity, defect distribution [9,10,11,12,13], and non-uniform loading of the cross-section. Furthermore, under higher stress amplitudes, rotating-bending specimens generally exhibited better fatigue resistance as compared to the axial specimens subjected to the same nominal stresses. At lower stress amplitudes, the difference in fatigue life became less obvious. This trend is suggested as the consequences of detrimental effects of multi-site crack initiation on both sides of the specimen under bending. Multiple propagating cracks were found on the fracture surface of the rotating-beam fatigue specimen, consistent with results reported previously in the literature.

Moreover, extending to the VHCF regime, various investigations have focused on three-point bending fatigue and cantilever bending fatigue research. Xu et al. [14] studied the microstructure and high cycle fatigue performance Ti46Al7Nb alloys and AZ31B magnesium alloy under three-point bending loading. Ma et al. [15] proposed a multi-area fatigue damage model of composite honeycomb sandwich panels under a three-point bending load. Yoshihiko et al. [16] evaluated the fatigue behavior of AZ31 magnesium alloy using the single-crystal micro cantilever specimen. Toshifumi et al. [17] studied the effect of grain orientation on the fatigue behavior in the micro cantilever of AZ31 magnesium alloy. Menzemer et al. [18] researched the fatigue and fracture responses of cantilevered luminaire structures made from 6063 aluminum alloy. However, the studies mentioned above that lead to fatigue failure in bending loading are relatively LCF or HCF. Plenty of results had proved that the fatigue failure behavior is different in LCF or HCF compared to in VHCF. Even though many attempts had been made in high-frequency loading to reach bending cycles to the very high cycles, a systematic study of such conditions has yet to be achieved due to the shortcomings of current experimental study methods, heating, transgression from the gauge section, and friction failure [19].

Given the limited results of existing research on bending fatigue at the VHCF regime, a new approach to ultrasonic bending fatigue is proposed in this work. The specimen is designed as a thin plate similar to a blade with a linear loading. Moreover, the second-order flexural vibration modal has been suggested with a frequency close to 20 kHz. In order to monitor the stress amplitude of the specimen and accurately record the fatigue life of the specimen in real-time, a laser-based measurement system is developed. The curvature of the sample is measured by a laser to obtain the stress amplitude. A cross point of S-N curves from bending and axial loading was obtained when fatigue life surpassed 10^7^ cycles.

## 2. Pure Bending Test Method

### 2.1. Composition of the Experimental System

An ultrasonic fatigue test instrument is indeed a resonant system, which is composed of an excitation power supply, a piezoelectric transducer, a horn, and the specimen sequentially assembled in series by fastening connections. The mechanical vibration generated by the piezoelectric actuator is meant to reproduce a pure sine wave with a frequency of 20 ± 0.5 kHz [20,21].

**Figure 1:**
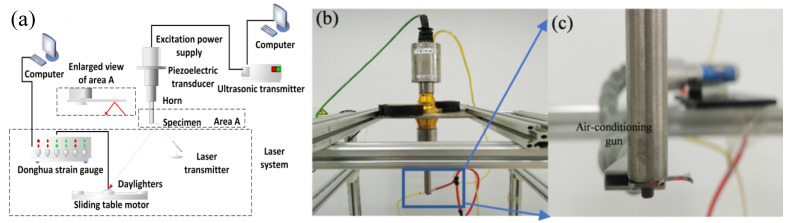
Ultrasonic bending fatigue vibration: (**a**) Schematic diagram of ultrasonic fatigue system; (**b**) Installation of ultrasonic bending equipment; (**c**) transition of a longitudinal wave to a transverse wave.

As shown in Figure 1a,b, the load control of the test piece connected to the piezoelectric transducer through a horn was realized by controlling the magnification of the computer system and the inputting. The displacement amplitude of longitudinal vibration was adjusted through the electrical signal into a mechanical displacement signal. Accordingly, the input voltage is embodied as the value of the input amplitude ratio in the control system [22,23].

At the bottom of the horn, in Figure 1c, the specimen was fixed similarly to a cantilever beam that was perpendicular to the vertical horn. This type of connection allows the displacement to transit from longitudinal loading on the horn to transverse loading on the specimen. Because of the transverse wave, the reversible curvature occurred on the test piece and makes the part of the specimen in the cyclic bending with a stress ratio of R = −1 in the frequency of 20 kHz.

Notably, an ultrasonic bending test also works in the harmonic vibration so the specimen must be customized in a geometry that obeys the free vibration model around 20 kHz.

### 2.2. Principle of Specimen Design for Ultrasonic Bending

Liu et al. introduced a design method for ultrasonic bending fatigue sheet specimens [22]. Further, it is known from the research that, based on vibration theory and the stress wave principle, the mode shape formula and frequency formula of free vibration is as follows:

(1) $$\cos a_{n}l_{n}\cos ha_{n}l_{n}\;=\;1$$

(2) $$\omega_{n}={\left(a_{n}\right)}^{2}\sqrt{\frac{Eb^{2}}{12\rho }},$$

where *l_n_* represents the length of *n*-order bending specimen, *ρ* represents the density, *b* represents the thickness of the equal section of the specimen, *E* is the elastic modulus, *a_n_* is the constant corresponding to the mode shape order, and $\omega_{n}\;$ is the angular frequency.

To compromise the wave transition section without deflection, which promises neither heating generation nor friction at contact points, pure bending of the thin plate specimen in this work is designed in the second-order of bending. Therefore, the length of the second-order bending specimen can be calculated from Equations (1) and (2), i.e.,

(3) $$l_{2}=0.1683{\left(\frac{Eb^{2}}{f^{2}\rho }\right)}^{\frac{1}{4}},$$

where *f* represents the resonance frequency.

For this particular resonant frequency of 20 kHz, the sample is calculated specifically for this natural frequency. According to the transverse wave elastic, the given geometry, and material properties, the maximum stress at the gauge section of the experimental sample during resonance is calculated as follows:

(4) $$\sigma {\left(x\right)}_{\max}=\;\frac{Eb\emptyset^{\prime \prime }\left(x\right)}{2},$$

where $\emptyset^{\prime \prime }\left(x\right)\;$ is the second-order derivative of the mode shape function, and $\sigma {\left(x\right)}_{\max}$ is the maximum stress amplitude. Therefore, the size of the specimen is preliminarily designed and determined according to the specific natural frequency and the required modes of vibration.

### 2.3. Specimen Final Design with Numerical Simulation

The theoretical formula provides only the basic shape of ultrasonic bending specimens. Two issues remain to be considered. Firstly, the specimen and the horn were fastened by a pin-hole type connection, which introduced the stress concentration at the connection section and may crack earlier than the gauge section by bending fatigue loading. An hourglass form with surpassing stress amplitude was obligate in the designing process to ensure the fatigue crack takes place at the gauge section. Secondly, to control the stress amplitude through the vibration displacement, the precise calculation of the stress–displacement coefficient ratio Cs was needed before launching a test. To meet the above requirements as well as the resonance frequency for running the ultrasonic bending fatigue test, finite element analysis was applied by ABAQUS for the final design of the specimen [22].

The material adopted in this work was Ti6A14V, with a thickness of 1.27 mm, a density of 4.5 g/m^3^, an elastic modulus of 110 GPa, and a Poisson’s ratio of 0.35. The performance of the specimen was simulated with the assumptions of homogeneous and linear elasticity. 

In the case of a single pin-hole type connection, the diameter of the hole can be increased to maintain sample stability. The increase in the hole diameter can raise the width of the gasket, which will affect the curvature of the test piece and cause a significant stress concentration subsequently. Moreover, the plane rotation constraint cannot be restricted effectively. Two perforations with a radius of 1.5 mm were employed at the connection section, and the test piece was connected through the pin-horn with the ring gasket and bolts. The stress concentration was enlarged by about 2.2 to 2.3 times due to the pin-holes connection.

**Figure 2:**
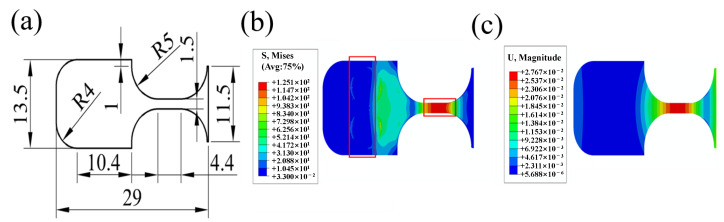
Specimen sketch and simulation result at the resonant frequency. (**a**) Dimensions of bending test sample; (**b**) Von-Mises stress field; (**c**) out-plan displacement field.

As shown in Figure 2a,b, the right part of the specimen was set as an hourglass form to ensure the maximum stress amplitude was located at the gauge section of 4.4 mm, so that the stress amplitude at the gauge section was about 2.5 times greater than the connection section and to guarantee the crack first occurring at the gauge section. Further, a chamfer with a radius of 4 mm was designed on the edge of the test piece, hence the frequencies of the adjacent modes of the test piece could be effectively separated to avoid the unexpected mutual vibration of multiple modes of the test piece. As indicated in Figure 1c, the maximum displacement occurred at the gauge section, and the vibration shape of the sample was in the second-order bending mode.

**Figure 3:**
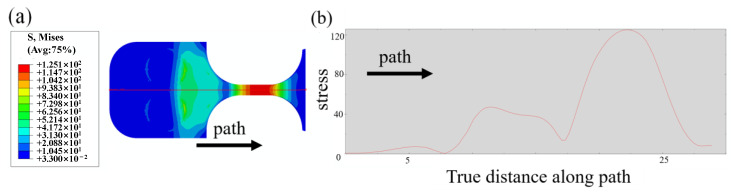
Stress amplitude along the axial of the sample. (**a**) Schematic diagram of uniaxial resonance stress path; (**b**) stress amplitude along the path with 1 µm given displacement.

Figure 3 presents the stress distribution of an ultrasonic bending fatigue specimen. Figure 3b plots the stress variation along the axial path marked in Figure 3a. The maximum stress in the section was about 125 MPa with an input of 1 μm displacement amplitude, thence the stress–displacement coefficient ratio Cs was about 125 MPa/μm. The simulated results, after calibration, were used to control the stress amplitude in the following ultrasonic experiments.

Moreover, severe local heating and insufficient cooling existed in the loading area, significantly impacting the specimen’s elastic modulus and yield strength, affecting the specimen’s fatigue strength. To make matters worse, the fretting fatigue caused by the fretting wear of the sample was not negligible. Additionally, since it was challenging for the cantilever beam to deliver effective lateral restraint on the test piece in the lateral direction [23], the test piece could produce a sizeable lateral displacement during resonance. In the cantilever beam bending experiment, the specimen’s fatigue failure occurred in the loading area, making the fatigue failure factors vary. Thus, it is challenging to analyze the cause of fatigue fracture of the specimen scientifically.

Therefore, the pure bending method with R = −1 is proposed in this work, where *R* represents the stress ratio of the sample during vibration, that is, $R\;=\;\frac{\sigma_{\min}}{\delta_{\max}}$. In addition, the bolts were used to connect the specimen to the ultrasonic equipment through double holes. The specimen was subjected to a linear loading constrained by large lateral displacement and rotation angle, to make the fatigue failure eventually occur in the experimental section.

## 3. Monitoring of the Fatigue Test

### 3.1. Stress Measurement 

**Figure 4:**
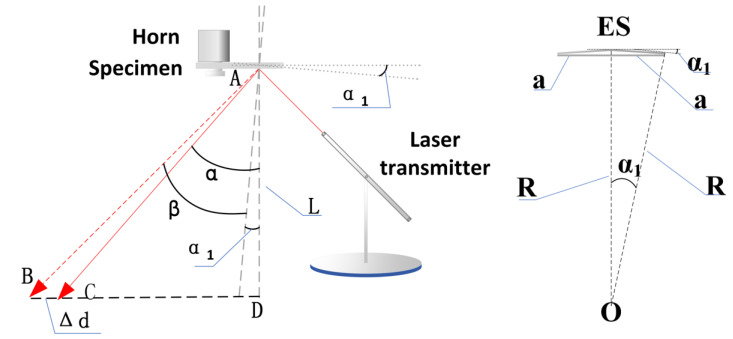
Schematic diagram of laser curvature measurement.

Stress measurement for ultrasonic fatigue is a great challenge, along with other accelerated fatigue loading. Firstly, the conventional strain gauges exhibit degradation in long-time fatigue service. Secondly, stress amplitude in VHCF is generally smaller than yield stress to some extent, so strain measurement by some other technique, such as digital image correlation (DIC), results in a large tolerance and difficult responses such as a high loading frequency of 20 kHz. Laser vibrometry is widely applied to detect vibration displacement based on the Doppler effect. With the given deformation model, the stress variation could be calculated from displacement. In this work, however, a low-cost stress measurement was developed with a laser projector to adapt the stable bending vibration. As shown in Figure 4, the laser was emitted from a point projector. After reflecting from the gauge section, the light was projected to a slidable photoresistor which revealed the deflected angle at the gauge section with the sliding distance. Accordingly, the real-time change of the voltage was recorded by the Dynamic Signal Test and Analysis System (DH5922D), and the moving distance of the laser during excitation could be received. A comparison was made between the slop and deflection of the light path caused. It was found that the light path by the deflection of the gauge section was extremely tiny so it could be ignored. Thus, the curvature formula can be simplified as follows:

(5) $$\frac{L}{\Delta d}=\frac{cos^{2}\alpha }{tan2\alpha_{1}}-sin\alpha cos\alpha$$

(6) $$\tan\alpha =\frac{L_{CD}}{L},$$

where *Δd* is the light path from the laser reflection point during excitation, *L* is the vertical distance from the test piece to the plane of the photoresistor, *α* is the laser reflection angle when non-resonant, *α*_1_ is the maximum value of the rotation angle of curvature while bending vibration, and *L_CD_* is the horizontal distance from the laser projector to the slidable photoresistor which marks the position of highest absorption after light reflected from the gauge section.

Because $\rho =\frac{a}{\alpha_{1}}$, $\frac{1}{\rho }=\frac{M}{EI}$, and $\sigma =\frac{My}{I}$, $y_{\max}=b$/2, the stress amplitude $\sigma_{a}$ is calculated accordingly as follows:

(7) $$\sigma_{a}=\frac{E\alpha_{1}b}{2a},$$

where *b* is the thickness of the test piece.

**Figure 5:**
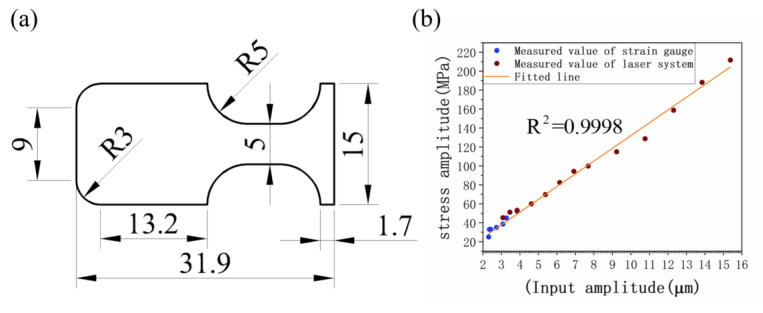
Laser measurement reliability verification: (**a**) Laser measurement standard sample size; (**b**) laser measurement data.

In order to prove the above method of stress amplitude measurement, the following experiments were conducted to verify the feasibility of the laser. For the reason of reducing the degradation of gauge sensitivity ratio from fatigue and facilitating strain gauge attachment on the sample surface, a particular sample that had a width of 5 mm, as in Figure 5, was used for the stress calibration and method validation. Simultaneously, a laser-photoresistor system was used to measure the curvature of the gauge section and to obtain the stress amplitude.

Results from the strain gauge and laser projector at different longitudinal vibrations are shown in Figure 5b. The stresses measured by the strain gauge and laser-photoresistor system are approximately equal for input displacement amplitudes from 3 to 4 μm. Furthermore, the overall and the overlapped part of the data fit well, indicating the reliability and rationality of the laser measurement method. Additionally, the slope of the fitting line corresponds to the stress–displacement coefficient ratio Cs which was compared with numerical simulation for its calibration. Notably, stress amplitude measured by a laser-photoresistor system can only apply to the stable vibration but not the arbitrary vibration and frequency detection.

### 3.2. Temperature Monitoring 

With an extreme loading frequency, an ultrasonic vibration would generate heat by accumulating friction in each cycle because of the inconsistent harmonic vibration under constrained conditions [24,25,26,27,28]. Although the novel method of second-order harmonic vibration, which ideally permits an angle of rotation equal to zero at the connection section, would help to decrease the heating generation, the absolute increase of temperature cannot be avoided because of friction at the pin-hole type connection and the microscopic irreversible deformation. Therefore, additional cooling of compressed air may be added to reduce the temperature of the specimen.

**Figure 6:**
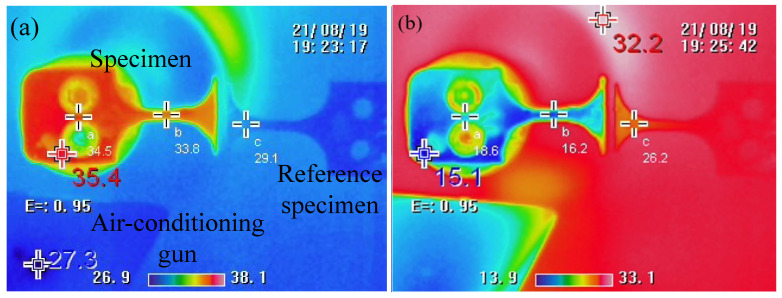
Thermal image of the specimen during resonance: (**a**) Without air-cooled; (**b**) air-cooled.

An infrared image in Figure 6 shows the case of resonance without air-cooling, where the temperature of the specimen is higher than the ambient temperature of 29.1 °C. The maximum temperature appeared around the pin-hole connection and is about 2 °C higher than it is at the gauge section. The double-hole design results in a small contact area, with no apparent frictional heating. In the case of resonance with air-cooling, Figure 6b shows that the temperature of the specimen is lower than the ambient temperature of 26.2 °C. The temperature on the specimen is maintained in ambient conditions throughout the period of VHCF testing, which was recorded and monitored by the infrared camera. Additionally, as indicated in Figure 6, the specimens made of TI6AL4V have no severe damping heat under high-frequency resonance. Thus, the increasing temperature factors that cause the fatigue failure of the specimen are ignored in this work.

## 4. Experimental Results and Discussion

### 4.1. S-N Curve and Fracture Data Statistics

**Figure 7:**
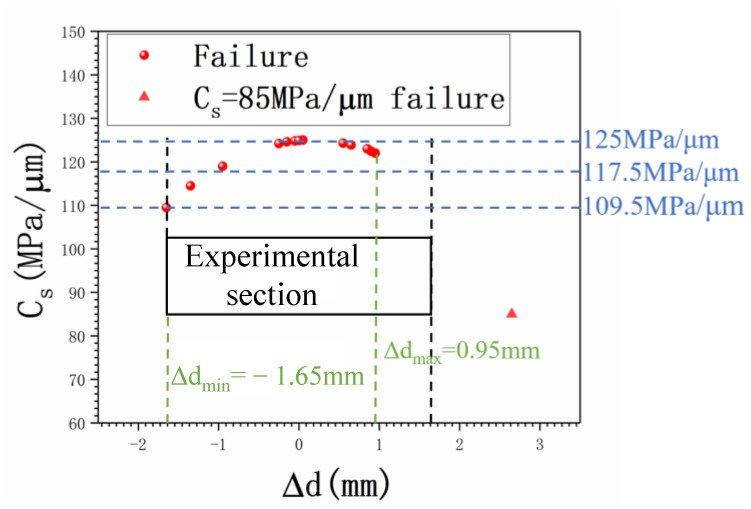
Statistics diagram for the fatigue failure.

As well as in the ultrasonic push–pull fatigue with the classic hourglass form specimen, fracture positions do not always occur at a maximum stress section which is mainly caused by the microstructural sensitivity in the VHCF regime. Therefore, amendment of stress amplitude is usually conducted based on the fracture position according to the stress gradient that existed along the axial of the specimen, as shown in Figure 3b and Figure 7. In the current study, a serial test consisting of 14 specimens was run to fatigue failure at different nominal stress amplitudes. After the fatigue, the exact failure position was precisely measured for each test, and the Grubbs criterion was applied for the gross error analysis before stress amendment. Accordingly, the confidence interval of 14 failure points is from 91.4 MPa/μm to 125 MPa/μm according to the Grubbs criterion where the confidence probability P equals 0.99, and Grubbs critical value G_99_ (14) equals 2.659. Thus, the data points *C*_s_ = 85 MPa/μm beyond the interval should be excluded. The confidence interval for the remaining 13 data points is from 109.3 MPa/μm to 125 MPa/μm where the confidence probability of P equals 0.99, and the Grubbs critical value of G_99_ (13) equals 2.607. Furthermore, 13 points are all within the error range of 6%. Therefore, considering the above issues comprehensively, a final S-N curve is obtained by equivalent stress gradient, i.e., $\sigma_{a}=k\sigma$, where *k* is the influence coefficient of stress gradient for stress amendment.

**Figure 8:**
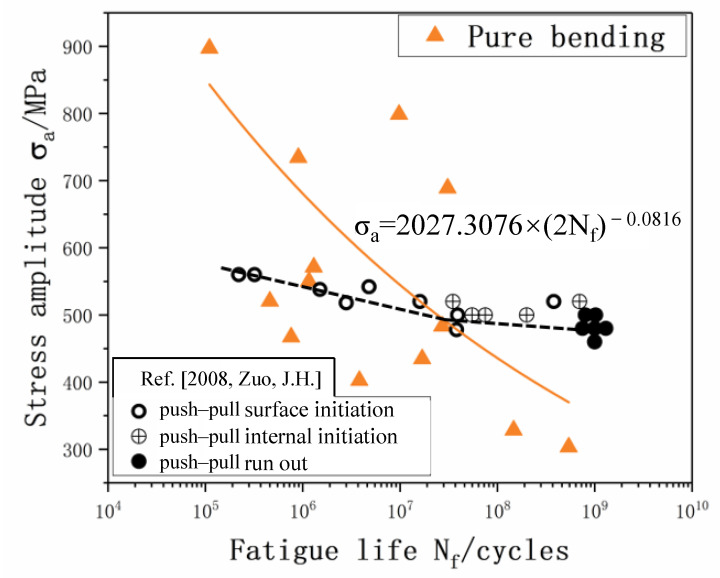
S-N curves of ultrasonic bending fatigue in comparison to the axial fatigue in literature.

The S-N curve of TI6AL4V obtained from the pure bending experiment with *R* = −1 is a continuous decline type known from Figure 8, where the bending fatigue stress under 10^9^ cycles is down to 350 MPa. Below 10^7^ cycles, as indicated in Figure 8, the pure bending fatigue stress is higher than that of the uniaxial push–pull fatigue from the literature [29]. That is, due to the influence of the loading method, the stress gradient of the bending specimen causes the average stress amplitude and control volume for internal defects to be lower than uniaxial loading in which the stress is equal in the cross section [30,31,32]. Therefore, the fatigue failure of the specimen requires a higher stress amplitude level, which increases the fatigue stress for the cyclic life below 10^7^ cycles. On contrary, the bending fatigue stress is lower than the uniaxial fatigue stress in the VHCF (*N*_f_ > 10^7^) regime. This phenomenon is rarely reported in previous studies in the literature. However, a tendency of intersection between bending fatigue curve and uniaxial fatigue curve is generally found when comparing the effect of loading type during a fatigue test. Because of the lack of sufficient data, the reason for this change is yet to be fully understood. A possible reason suggested in this work is that uniaxial fatigue samples are not affected by the stress gradient, with the crack initiation’s internal nucleation in VHCF leading to a longer life of uniaxial fatigue samples. On the other hand, due to stress gradients, the crack initiation site in bending fatigue will not alter with the change of stress amplitude and always results in a surface crack nucleation. Thus, the bending fatigue stress is lower than the uniaxial fatigue stress in VHCF. Furthermore, the discreteness of pure bending fatigue is higher than that of push–pull fatigue. The reason is that, since the fatigue life and corresponding stress amplitude show a competitive relationship with the defects in the material and the volume of material under larger fatigue amplitude of gradient stress, the bulk in its localized stress amplitude significantly influences the fatigue property. The lower the stress amplitude, the slower the gradient slope in the cross-section, which further decreases the volumetric effect of the stress gradient.

### 4.2. Fracture Morphology

**Figure 9:**
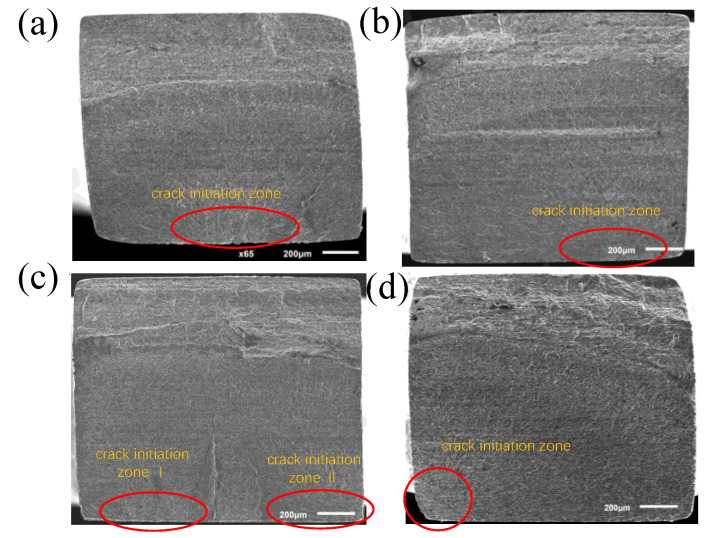
Pure bending fracture morphology: (**a**) σ_a_ = 555 MPa, *N*_f_ = 3.82 × 10^6^ (**b**) σ_a_ = 435 MPa, *N*_f_ = 1.69 × 10^7^; (**c**) σ_a_ = 335 MPa, *N*_f_ = 1.47 × 10^8^; (**d**) σ_a_ = 305 MPa, *N*_f_ = 5.41 × 10^8^.

The fracture morphology was observed using a scanning electron microscope (SEM) as shown in Figure 9 for the sample tested with the novel ultrasonic bending fatigue. It shows a similar feature of fractography taken from the flexural fatigue sample studied in the literature [33,34,35,36]; i.e., due to the stress gradient, single or multiple crack initiation sources nucleate on the surface, which expands from the outside to the inside. Moreover, the crack is a river-like shape during the expansion, and the expansion direction is about 45° inclined.

**Figure 10:**
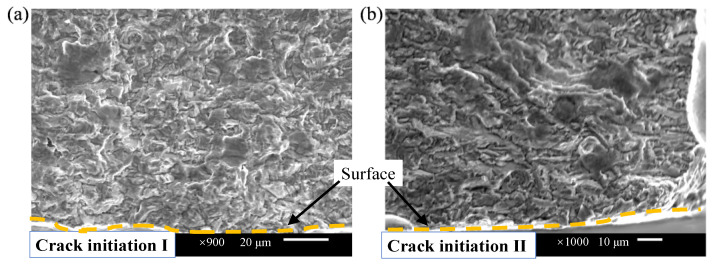
Pure bending fracture morphology: (σ_a_ = 335 MPa, *N*_f_ = 1.47 × 10^8^) crack initiation morphology: (**a**) initiation I; (**b**) initiation II.

Unlike the finding in the fracture from axial fatigue [37,38], facets at the initiation area are inconspicuous in the fracture surface of bending fatigue specimens as shown in Figure 10. In the uniaxial push–pull fatigue fracture process [39,40,41,42], the stress distributions were uniform and the crack initiation distribution was random. Indeed, the crack in uniaxial fatigue fracture may nucleate on the surface or inside by correlating the subscale damages and exhibits a “fish-eye” shape under a very high cycle load. However, due to the influence of the stress gradient, the bending fatigue crack originates from the surface, and no obvious “fish-eye” morphology is found (Figure 9). Moreover, the crack propagates along the path from the high-stress level to the low one under the bending stress gradient.

**Figure 11:**
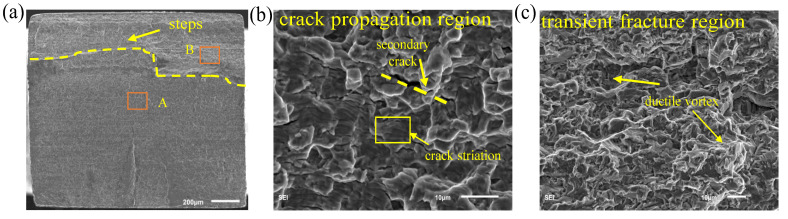
Fractography of sample failed by bending: (**a**) Pure bending (σ_a_ = 335 MPa, *N*_f_ = 1.47 × 10^8^) crack morphology; (**b**) enlarged image of area A crack propagation region); (**c**) enlarged image of area B (transient fracture region).

As can be seen in Figure 11b, the crack propagation region can reflect the corresponding fatigue failure characteristics such as secondary cracks and fatigue bands. Figure 11a,c shows the overall fracture topography and part of the transient fracture region contains a large number of ductile vortices and steps due to the quick fracture.

The stress gradient generated by the bending causes the maximum stress to appear on the surface of the specimen, which leads to the nucleation of the crack source on the surface of the specimen. Therefore, for the purpose of fatigue resistance in the design of core components serving aviation, aerospace, ships, and automobiles, the load environment of components is suggested to consider not only the loading type but also the slop of stress gradient. Moreover, unlike the switch of initiation site from the surface to the inside in the VHCF regime, surface treatment [43] to increase fatigue life likely also works for bending fatigue deformation even beyond VHCF. Thus, the work in this paper provides an alternative method for performing bending fatigue testing in the VHCF with the advantages of less time and less material consumption.

## 5. Conclusions

In this work, a novel model for the pure bending loading ultrasonic fatigue test research method is proposed based on the development of an ultrasonic platform, and the obtained data are statistically analyzed. SEM is used to observe the fracture characteristics of the uniaxial bending fatigue specimen. The main conclusions are as follows:

(1) Fatigue failure occurs at the gauge section, and the equivalent S-N curves are obtained after the effects of the crack section are taken into account. To confirm the validity of the novel method, the fracture results and fracture characteristics in this work are in agreement with those existing in the literature;
(2) The harmonic model of second-order vibration provides an angle of rotation equal to zero, which significantly reduces the heat generation from friction at the connection area;
(3) Following the tendency found in the LCF and the HCF, the S-N curves have an intersection between bending fatigue and uniaxial fatigue in the VHCF regime. However, unlike the uniaxial fatigue, the inner crack invitation vanished in the bending case for the test material.

As an insufficient result for different materials, the above features, such as the intersection of the S-N curve, may not be applied to other metals. Besides, the ultrasonic bending fatigue test in this study currently only works for thin sheet plates less than 2.5 mm thick. A thick sample requires an extremely difficult geometric design due to harmonic resonance and would increase heat generation.

## Data Availability

Not applicable.
